# Dental Caries, and Supragingival Plaque and Calculus among Students, Tanga, Tanzania

**DOI:** 10.5402/2012/245296

**Published:** 2012-03-12

**Authors:** L. C. Carneiro, M. N. Kabulwa

**Affiliations:** ^1^Department of Restorative Dentistry, Muhimbili University of Health & Allied Sciences, P.O. Box 65451, Dar es Salaam, Tanzania; ^2^Department of Preventive & Community Dentistry, Muhimbili University of Health & Allied Sciences, P.O. Box 65014, Dar es Salaam, Tanzania

## Abstract

The prevalence of dental caries and supragingival plaque and calculus in 785 secondary schools students was assessed. More than half (53.6%) of the students were caries-free, and the majority of those with dental caries experience were aged 14–17 (68.1%) and females (53%). Mean DMFT was 1.26, with mean D-component of 1.05, and molars were most affected. Most students had supragingival plaque (74%) and calculus (56.9%) and more so in males than females (*P* > 0.05). Less than half of the students had experience of dental caries and those with caries were mostly females and of the younger age group. The low DMFT was contributed to the D-component, and molars were the tooth type most affected.The majority of students had supra-gingival plaque and calculus and more so in males than females.

## 1. Introduction

Oral health is now recognized as equally important in relation to general health [1], and absenteeism from work associated with dental problems and the undocumented effects on the level of performance of children in class are now recognized as problems of public health and socioeconomic concern [2]. The major oral health problems around the world are generally considered to be dental caries and periodontal diseases [3]. Periodontal diseases are among the most widespread diseases in mankind [4]; an estimated 60–90% of school children worldwide and most adults have experienced dental caries [5]. Within the Tanzanian community oral diseases are common [6–8], and the quality of life of Tanzanians affected by dental caries and periodontal disease has been documented [9, 10]. Previous studies show that most individuals seek dental care with complaints of pain mainly because of tooth ache related to dental caries [11, 12]. It has also been reported that the prevalence of severe type of periodontal disease is very low and affects only a minority [13], even though poor oral hygiene is a problem of the majority [7]. Determining the prevalence of dental caries and supragingival plaque and calculus of secondary school students in Tanga, Tanzania, will provide baseline data that is necessary for planning of intervention programs in schools. Preventing or reducing the prevalence of dental caries and periodontal disease among students will assist in improving their quality of life.

## 2. Study Population and Methods

This cross-sectional study conducted between September and November, 2010, assessed the prevalence of dental caries, supragingival plaque and calculus of 785 secondary school students in Tanga Region, Tanzania. Tanga Region has eight districts (Tanga, Lushoto, Korogwe, Muheza, Mkinga, Pangani, Handeni, and Kilindi); two districts, namely, Tanga and Lushoto, were conveniently chosen for this study.

A total of eight schools were conveniently chosen for the study, four schools from each district of Lushoto and Tanga. From each school, a sample size of hundred students, fifty students from form II (25 boys and 25 girls) and fifty students from form III (25 boys and 25 girls) were randomly selected by the teachers on duty. Estimated sample size was 800 students with a response rate of about 98%, as students who did not participate in Phase I of the study [14] were omitted.

Following written consent from each student, examination of each student's dentition was performed using natural light, a dental explorer, and dental mirror while lying on a bench, and examiner seated behind the subjects head. Using the World Health Organization (WHO) diagnostic criteria [15], the number of decayed, missing and filled teeth (DMFT) was recorded and when the examiner was in doubt no caries was recorded. For analysis students were categorized according to their caries experience, those without caries experience (DMFT = 0; absence of a decayed, missing tooth/teeth due to caries, or filled tooth/teeth) or those with a caries experience (DMFT ≥ 1; presence of one or more decayed, missing tooth/teeth due to caries, or filled tooth/teeth).

Scoring of supragingival plaque and calculus was done in accordance to a modified version of the WHO diagnostic criteria [15]. The criteria was modified into two scores (absent = 0 and present = 1). The supragingival surfaces of the index teeth (tooth 16, 11, 26, 36, 31, and 46) were assessed for presence/absence of supragingival plaque and calculus. A score = 1 was given when visible plaque remnants were present on any of the index teeth, and a score = 0 was given when there were no visible remnants present on any of the index teeth. In doubtful instances plaque was scored as absent. When supragingival calculus was observed on any of the supragingival surfaces of the index teeth it was scored present = 1, and when no deposits were observed on any of the supragingival surfaces of the index teeth it was scored absent = 0. Subjects were then categorized to be plaque-free or calculus-free (score = 0) when there was absence of supragingival plaque or calculus in all of the index teeth and with plaque or calculus (score = 1) when there was presence of supragingival plaque or calculus in one or more of the index teeth.

Infection control was maintained by the use of gloves and masks by the examiners, and the dental mirrors were placed in Steranios 2% (LOT 119113. ANIOS laboratories, Pave du Moulin, France) for 10 minutes, rinsed with water and sterilized using a pressure cooker and gas stove prior to use.

The interexaminer variability of the two examiners was assessed by reexamining 10% of the subjects in four schools (Tanga = 2; Lushoto = 2), and kappa values ranged from 0.64 to 1.0 for dental caries, 0.14 to 0.33 for supragingival plaque and 0.1 to 0.39 for supragingival calculus.

Data collected was coded, and using the SPSS statistical package analysis was performed. Chi-square tests were used to determine the level of statistical significant difference at *P* < 0.05. Ethical clearance was obtained from the Director of Research and Publications, Muhimbili University of Health and Allied Sciences, Dar es salaam, Tanzania.

## 3. Results 

A total of 785 secondary school students were analyzed with a representation of about twelve percent from each of the eight schools. The age range was 14 to 22 years and the mean age was 16.93 years. There were more students from Tanga district (50.2%) than Lushoto district (49.8%). Females (50.3%) were more than males (49.7%), and age group of 14–17 years was the majority (69.8%) (Table 1).

Shown in Table 2 is the distribution of students according to their dental caries experience by district, age, and sex. More than half (53.6%) of the students had no experience of dental caries and among those who had experience of dental caries majority were from Lushoto. Students aged 14–17 had a higher dental caries experience (68.1%) than those aged 18 and above, and females were observed to have a higher dental caries experience (53%) than their counterparts.

Of the students (*n* = 364; 46.4%) with dental caries experience (DMFT), the majority (*n* = 334; 42.5%) had one or more decayed tooth/teeth (D-component), few (*n* = 105; 13.4%) had one or more teeth missing due to caries (M-component), and a small number (*n* = 5; 0.6%) had one or more teeth filled due to caries (F-component). The students mean DMFT was 1.26, with mean D-component of 1.05, mean M-component of 0.20, and mean F-component of 0.0089. The number of decayed teeth per person ranged from 1 to 27, the number of missing teeth ranged from 1 to 6, and the number of filled teeth ranged from 1 to 3.

Shown in Table 3 is the distribution of the decayed teeth of students by tooth type. Of the 823 decayed teeth of students the majority were from the lower jaw (*n* = 438; 53.2%) and the tooth type most affected by dental caries was molars and the least affected was the anterior teeth. There were statistically significantly more molars in the lower jaw than upper jaw affected by caries, and upper premolars and anterior teeth were statistically significantly more affected than the lower premolars and anterior teeth (*χ* ^2^ = 80.08; *df* = 2; *P* = 0.001).

Table 4 shows the distribution of students supragingival plaque score by district, age, and sex. The proportion of students with supragingival plaque (*n* = 581; 74%) was much higher than those without plaque (*n* = 204; 26%). There were statistically significantly more students from Lushoto with supragingival plaque compared to those from Tanga (*P* < 0.01).

There was no statistical significant difference between the two age groups in regard to occurrence of supragingival plague. Males with supragingival plaque were statistically significantly more that their counterparts.

Of the 74% (*n* = 581) of students with supragingival plaque, sextant 1 (*n* = 432; 74.4%) was the most affected followed by sextant 3 (*n* = 426; 73.3%), sextant 2 (*n* = 389; 67.0%), sextant 5 (*n* = 359; 61.8%), sextant 4 (*n* = 317; 54.6%), and sextant 6 (*n* = 295; 50.8%). The mean number of sextants with plaque was 2.83.

The distribution of students supragingival calculus score by district, age group, and sex is shown in Table 5. The proportion of students with supragingival calculus in at least one sextant was more (56.9%) than the proportion of students who were calculus-free. There were statistically significantly more students in Tanga district with supragingival calculus than those in Lushoto district. The proportion of students with supragingival calculus did not show any statistical significant difference by age or gender.

Of the four hundred and forty-seven students with supragingival calculus the majority had calculus in sextant 5 (*n* = 315; 70.5%), followed by sextant 3 (*n* = 207; 46.3%), sextant 1 (*n* = 186; 41.6%), sextant 4 (*n* = 108; 24.2%), sextant 6 (*n* = 92; 20.6%), and sextant 2 (*n* = 46; 10.3%). The mean number of sextants with calculus was 1.22.

## 4. Discussion

Schools remain an important setting offering an efficient and effective way to reach over 1 billion children worldwide and, through them, families and community members [16]. This study assessed the prevalence of dental caries and supragingival plaque and calculus in secondary school students in Tanga, Tanzania, so as to provide baseline data for planning intervention programs that will assist in reducing the prevalence of dental diseases.

Limitations of the convenient sampling method used in this study are acknowledged and discussed in phase one of the study [14]. Although modified, the World Health Organization (WHO) Oral Health diagnostic criteria [15] used allow for comparisons of findings with other studies. Also the periodontal status of students that was assessed on presence/absence of supragingival plaque and calculus only may be an added limitation. Written consent was obtained from the students themselves and not parents as students were above the age of 14 years.

Results from this study revealed that the proportion of students who were caries-free was slightly over fifty percent implying that every second child had a caries experience, and these findings were similar to those reported in another study conducted among 12-year-old school children in Dar es salaam, Tanzania [17]. Contrastingly, a higher proportion of early adolescents were reported to be caries-free in Kilwa district, Tanzania [18]. The difference in proportion of disease between the two different locations in the same country could be related to urbanization as documented by Thorpe [3].

In this study the proportion of females with dental caries was observed to be higher than males, and similar findings have been reported by other researchers in Tanzania [17, 19, 20], Nigeria [21] and Kenya [22]. Findings from this study portray that females carry the biggest burden of dental caries that could be explained by their easier access to food supplies and frequent snacking during food preparation.

In this study the mean DMFT of 1.26 (mean D-component of 1.05, mean M-component of 0.20, and mean F-component of 0.0089) reported is lower than the WHO target of mean DMFT of 1.5. [23], but higher than other studies done in Tanzania [20, 24, 25]. The low mean DMFT reported could be a reflection of the low economic status of most Tanzanians. Similar to other studies [20, 24] in Tanzania and other countries in Africa [21, 26, 27], the decayed component mainly contributed to the reported DMFT.

The tooth type most affected by dental caries in this study were the molars and similar findings was also reported by other researchers [20, 24, 28]. The anatomy and eruption time of molar teeth could be the probable explanation of why molar teeth are prone to dental caries. Although this study portrays that dental caries affects lower molar teeth more than upper molars, further research is necessary to establish which jaw tooth type is most affected by dental caries.

Another study done in Tanzania [24] reported no teeth with fillings; however, in this study the filled component reported was low indicating poor utilization of restorative care services by the population. If the “F” component of DMF-T has to be raised by 10% as stipulated by the Policy Guidelines for Oral Health Care in Tanzania (2002) [29], tooth extraction which is mostly provided should be reduced and conservation should be encouraged although many still have limited access to oral health services.

The reported high proportion of students with supragingival plaque and calculus may be an indication that oral hygiene practices are not adequately performed. Similar to the reported findings, the World Health Organization [30] also reported a high occurrence of gum disease among older children and adolescents, with 50% to 100% of 12-years-old children having the signs of gum inflammation. The higher percentage of males than females with plaque and calculus reported in this study is similar to findings reported by other researchers in Tanzania [20, 31].

Sextant five had the highest prevalence of supragingival calculus, and although it could be related to the presence of the lingual salivary duct, it could also be related to inadequate brushing of the affected surfaces.

## 5. Conclusion 

Less than half of the students had experience dental caries and those with caries were mostly females and of the younger age group. The low DMFT was contributed to the D-component, and molars were the tooth type most affected. majority of students had supragingival plaque and calculus and more so in males than females.

**Recommendations**

- More studies should be conducted in other regions for comparison.
- Males should be encouraged to practice oral hygiene.
- Intervention programs should focus on maintaining the reported DMFT by changing the decayed component to the filled component.
- Dental check-up should be conducted in schools regularly.

## Figures and Tables

**Table 1:** Percent distribution of students by district, age, and sex (*N* = 785) (percentages in parenthesis).

		District					
		Tanga		Lushoto		Total	
		*n*	%	*n*	%	*n*	%

Age group	14–17	283	(71.8)	265	(67.8)	548	(69.8)
(years)	18+	111	(28.2)	126	(32.2)	237	(30.2)
Sex	Male	193	(49.0)	197	(50.4)	390	(49.7)
	Female	201	(51.0)	194	(49.6)	395	(50.3)

Total		394	(50.2)	391	(49.8)	785	(100.0)

**Table 2:** Distribution of students according to their dental caries experience by district, age, and sex (*N* = 785) (percentages in parenthesis).

		Without caries experience		With caries experience		Total	
		*n*	%	*n*	%	*n*	%

District	Tanga	217	(51.5)	177	(48.6)	394	(50.2)
	Lushoto	204	(48.5)	187	(51.4)	391	(49.8)
Age group	14–17	300	(71.3)	248	(68.1)	548	(69.8)
(years)	18+	121	(28.7)	116	(31.9)	237	(30.2)
Sex	M	219	(52.0)	171	(47.0)	390	(49.7)
	F	202	(48.0)	193	(53.0)	395	(50.3)

Total		421	(53.6)	364	(46.4)	785	(100.0)

**Table 3:** Distribution of the decayed teeth of students by tooth type (*N* = 823) (percentages in parenthesis).

Location	Molars		Premolars		Anterior		Total	
	*n*	%	*n*	%	*n*	%	*n*	%

Upper jaw	254	(65.9)	72	(18.7)	59	(15.4)	385	(46.8)
Lower jaw	399	(91.1)	17	(3.9)	22	(5.0)	438	(53.2)

Total	653	(79.3)	89	(10.8)	81	(9.8)	823	(100.0)

**Table 4:** Distribution of students supragingival plaque score by district, age, and sex (*N* = 785) (percentages in parenthesis).

		Plaque free		With Plaque		Total	
		*n*	%	*n*	%	*n*	%

District	Tanga	119	(58.3)	275	(47.3)	394	(50.2)
	Lushoto	85	(41.7)	306	(52.7)**	391	(49.8)
Age group	14–17	148	(72.8)	400	(68.8)	548	(69.8)
(years)	18+	56	(27.2)	181	(31.2)	237	(30.2)
Sex	M	85	(41.7)	305	(52.5)**	390	(49.7)
	F	119	(58.3)	276	(47.5)	395	(50.3)

Total		204	(26.0)	581	(74.0)	785	(100.0)

Chi-square test: **P < 0.01.

**Table 5:** Proportion of students supragingival calculus score by district age and sex (*N* = 785) (percentages in parenthesis).

		Calculus-free		With calculus		Total	
		*n*	%	*n*	%	*n*	%

District	Tanga	145	(42.9)	249	(55.7)***	394	(50.2)
	Lushoto	193	(57.1)	198	(44.3)	391	(49.8)
Age group	14–17	235	(69.5)	313	(70.0)	548	(69.8)
(years)	18+	103	(30.5)	134	(30.0)	237	(30.2)
Sex	M	154	(45.6)	236	(52.8)	390	(49.7)
	F	184	(54.4)	211	(47.8)	395	(50.3)

Total		338	(43.1)	447	(56.9)	785	(100.0)

Chi-square test: ***P < 0.001.
